# The effect of anti-cancer and anti-tuberculosis treatments in lung cancer patients with active tuberculosis: a retrospective analysis

**DOI:** 10.1186/s12885-020-07622-6

**Published:** 2020-11-19

**Authors:** Mei Chai, Qingming Shi

**Affiliations:** Department of Oncology, Anhui Chest Hospital, Hefei, 230032 China

**Keywords:** Lung cancer, Tuberculosis, Prognosis

## Abstract

**Background:**

Lung tuberculosis (TB) and lung cancer have a complex relationship. Data concerning TB treatment in lung cancer patients are still incomplete. The aim of this study was to investigate the effects of anti-cancer and anti-tuberculosis treatments in lung cancer patients with active lung TB.

**Methods:**

In a retrospective cohort study, lung cancer patients with active lung TB were identified between January 2013 and December 2016. Age- and sex-matched lung cancer patients without tuberculosis were selected as control subjects. Anti-cancer and anti-tuberculosis treatments were administered according to the national guidelines. The clinical courses and responses of lung cancer patients with and without active lung TB were examined and compared.

**Results:**

A total of 31 consecutive lung cancer patients were diagnosed with active lung TB. Fifty-one lung cancer patients without TB were enrolled as control subjects. Most patients in the two groups were elderly, had advanced non-small cell lung cancer and had tumor burdens. The anti-cancer treatment completion rate and response rate were not different between two group (87.1% in TB treatment patients vs. 92.2% in lung cancer patients, 77.4% in TB treatment patients vs. 88.2% in lung cancer patients, respectively). The anti-tuberculosis treatment completion rate and success rate was 87.1 and 80.7%. The median survival times were not different between two groups (52 weeks in TB treatment patients vs. 57 weeks in lung cancer patients). The change in Karnofsky performance score was also not different between two groups. The most common side effect in TB treatment patients was liver injury (61.3%). The most serious side effect in TB treatment patients was leukocyte deficiency (9.7% in Grade 3). Both of side effects mentioned above were not different between two groups.

**Conclusion:**

Both anti-cancer and anti-tuberculosis treatments can be safely and effectively administered in lung cancer patients with active lung TB. Attention should be paid to the risk of tuberculosis in lung cancer patients in TB high-burden countries.

## Background

Lung cancer and tuberculosis (TB) represent major public health problems worldwide, especially in developing countries. In China, the annual numbers of new cases of lung cancer and tuberculosis were estimated to be nearly 3,804,000 cases in 2014 [1] and 889,000 cases in 2017 [2], respectively.

The possible relationship between lung cancer and lung TB has attracted attention for several decades. An increasing number of studies have demonstrated that lung TB is associated with an increased risk and mortality of lung cancer and vice versa [3–6]. However, data concerning TB treatment in lung cancer patients are still incomplete, except for studies involving a small number of patients and showing inconsistent results [7–9].

To investigate the effects of anti-cancer and anti-tuberculosis treatments in lung cancer patients with active lung TB, we performed a retrospective case-control study in a cohort of patients.

## Methods

### Setting

The study was performed at Anhui Provincial Chest Hospital, a tertiary referral hospital for TB in Anhui Province that has an intermediate incidence of active TB cases (58.4/100,000) [10]. The study was approved by the ethics committee of our hospital.

### Patient selection

Using the electronic patient data system, patients with lung cancer and active lung TB were screened from January 2013 until December 2016. Age-, sex- and cancer stage-matched control subjects were randomly selected from lung cancer patients without TB during the same period at Anhui Provincial Chest Hospital.

### The diagnosis of active lung TB and lung cancer

The diagnosis of active lung TB was made on the basis of sputum smear and/or culture [11]. Ziehl-Neelsen staining technique was adopted as the method of sputum smear microscopy. Liquid media with the Mycobacteria Growth Indicator Tube was used for sputum TB culture. Due to limited resources, drug susceptibility testing to TB was not determined. Molecular testing, such as line-probe assays and Xpert MTB/RIF assay, was not used. The diagnosis of cancer was confirmed by histopathological examination [12].

### The treatment of active lung TB and lung cancer

The treatment of lung cancer and TB was in accordance with national guidelines [11, 12]. In brief, anti-tuberculosis chemotherapy was initially administered with three drugs: rifampicin, isoniazid, and ethambutol for at least 6 months. Anti-cancer chemotherapy was administered with third-generation platinum-based regimens for non-small cell lung cancer or cisplatin plus etoposide for small cell lung cancer. No molecular targeted therapy or radiotherapy for lung cancer was given to any patient during the study period. Because of economic and medical underdevelopment during study period, DNA testing and molecular targeted therapies were not given to any patient. For fear of radiation pneumonitis, radiotherapy was not administered to patients. After lung cancer and/or TB diagnosis, corresponding treatments were administered as soon as possible. Whether or not to adjust the chemotherapy regimen and/or dosage to cancer and TB was determined by attending doctors according to the side effects.

### Assessment of chemotherapy outcomes

The responses to anti-cancer treatment were defined according to the response evaluation criteria in solid tumors (RECIST 1.1) [13] and accessed by chest computed tomography every two chemotherapy cycles. Since one month after the initiation of anti-tuberculosis treatment, sputum smear were performed on consecutive 3 days every month. The outcome of anti-tuberculosis treatment was defined according to the World Health Organization’s definitions [14]. The side effects of chemotherapy were graded using the National Cancer Institute Common Terminology Criteria for adverse events, version 4.0 [15].

### Statistical analysis

Continuous variables were expressed as the median (interquartile range), and differences between groups were analyzed using the Mann-Whitney test. Categorical variables were expressed as absolute values and percentages and were analyzed using the chi-square or Fisher’s exact tests. *P* < 0.05 was considered to be statistically significant. A statistical software package was used for the analyses (SPSS 16.0, SPSS, Chicago, USA).

## Results

During the study period, 31 consecutive lung cancer patients were diagnosed with active lung TB. The lung cancer control group included 51 age-, sex- and cancer stage-matched patients without TB.

### The clinical characteristics of the patients in two groups (Table 1)

The median ages in the two groups were approximately 65 years. The age of patients in the TB treatment group was slightly younger than that of the patients in the lung cancer group. Male patients comprised 94 and 84% of each group. Compared to the TB treatment group, the lung cancer patients had a higher body mass index (BMI).

**Table 1:** Comparison of clinical, laboratory and cancer characteristics between groups

variable	TB treatment n (%)	Lung cancer n (%)	*P* value
Demographic			
Age, years	62.1 (25.0–80.0)	65.4 (45.0–83.0)	0.151
Male, n (%)	29 (93.5)	43 (84.3)	0.220
BMI (kg/m^2^)	20.8	21.9	0.024
Smoker, n (%)	20 (64.5)	28 (54.9)	0.398
Laboratory			
Hypoalbuminemia, n (%)	15 (48.4)	12 (23.5)	0.020
Anemia, n (%)	12 (38.7)	17 (33.3)	0.627
Liver dysfunction, n (%)	5 (16.1)	8 (15.7)	0.958
Renal dysfunction, n (%)	7 (22.6)	12 (23.5)	0.923
ESR elevation, n (%)	12 (48.0)	13 (32.5)	0.218
Cancer			
Tumor burden, n (%)	28 (90.3)	42 (82.4)	0.328
Type			
Non-small cell, n (%)	25 (80.6)	37 (72.5)	0.635
Adenocarcinoma	10 (32.3)	17 (33.3)	
Squamous cell	15 (48.3)	19 (37.3)	
Large cell	0	1 (1.9)	
Small cell, n (%)	6 (19.4)	14 (27.5)	
Stage			
Not evaluable	6 (19.4)	5 (9.8)	0.269
I, n (%)	0 (0)	2 (3.9)	
II, n (%)	2 (6.5)	3 (5.9)	
III, n (%)	4 (12.9)	15 (29.4)	
IV, n (%)	19 (61.3)	26 (50.9)	
KPS	84 ± 8	86 ± 9	0.333

Hypoalbuminemia was defined as a serum albumin concentration < 35 g/L. Anemia was defined as a hemoglobin level < 120 g/L in women and < 130 g/L in men. Liver dysfunction was defined as the total bilirubin levels ≥21  μmol/L and/or prothrombin time index < 50% and/or serum alanineaminotransferase > 35u/L and/or serum aspartate aminotransferase > 35 u/L. Renal dysfunction was defined as serum creatinine ≥88 μmol/L. ESR (erythrocyte sedimentation rate) elevation was defined as ESR ≥ 100 mm/h. Tumor burden was defined as tumor was detected by chest computed tomography. *KPS* Karnofsky performance score

The laboratory findings, including anemia, liver and renal injury, and the erythrocyte sedimentation rate (ESR) were nearly similar between the two groups. However, the proportion of patients in hypoalbuminemia was higher in the TB treatment group than that in the lung cancer group.

The cancer type and stage were similar between two groups. Most patients had advanced non-small cell lung cancer (NSCLC), especially adenocarcinoma and squamous cell carcinoma and had tumor burdens.

### Clinical course and response to anti-cancer treatments (Table 2, Fig. 1)

The anticancer chemotherapy regimens and the overall treatment completion rates were similar between two groups. Most of patients in two groups received first-line chemotherapy regimens with two agents, especially gemcitabine or pemetrexed or etoposide plus platinum. The treatment completion rate was 87.1% in the TB treatment group and 92.2% in the lung cancer group. The cycles of chemotherapy were not different between two groups. Maintenance treatment was not administered to patents according to with national guidelines. The response rates to anti-cancer treatment were similar between two groups: patients in the stable disease were 70.9% in the TB treatment group and 78.4% in the lung cancer group. The median survival time and Karnofsky performance score (KPS) changes were similar between two groups. The median survival times of patients in the TB treatment group and lung cancer group were 52 weeks and 57 weeks, respectively. The survival times of NSCLC and SCLC patients with stage III/IV were also not significantly different. Most patients in two groups were in the KPS stable state (87.1% in the TB treatment group and 78.4% in the lung cancer group).

**Table 2:** Comparison of anticancer therapies between groups

variable	TB treatment n (%)	Lung cancer n (%)	*P* value
treatment line of anti-cancer chemotherapy			
Adjuvant treatment	3 (9.7)	9 (17.6)	0.056
First line	25 (80.6)	42 (82.4)	
Second line or later	3 (9.7)	0 (0)	
Chemotherapy regimen			
Single agent	5 (16.1)	10 (19.5)	0.308
Gemcitabine	3 (9.7)	2 (3.9)	
Pemetrexed	1 (3.2)	2 (3.9)	
Etoposide	0	4 (7.8)	
Paclitaxel	1 (3.2)	2 (3.9)	
Two agents	26 (83.9)	41 (80.5)	0.879
Gemcitabine plus platinum	12 (38.7)	15 (29.4)	
Pemetrexed plus platinum	7 (22.6)	14 (27.5)	
Etoposide plus platinum	6 (19.4)	10 (19.6)	
Paclitaxel plus platinum	1 (3.2)	2 (3.9)	
Cycles of chemotherapy	4 (3–6)	5 (4–6)	0.923
Treatment completion rate			
Completion	27 (87.1)	47 (92.2)	0.454
Active withdrawal	4 (12.9)	4 (7.8)	
Response			0.322
Complete response	0 (0)	1 (2.0)	
Partial response	2 (6.5)	4 (7.8)	
Stable disease	22 (70.9)	40 (78.4)	
Progressive disease	7 (22.6)	6 (11.8)	
Median survival (weeks)	52 (22–82)	57 (36–64)	0.505
Non-small cell (stage III/IV)	52 (26–82) (*n* = 22)	48 (32–64) (*n* = 29)	0.331
Small cell (stage III/IV)	22 (21–33) (*n* = 6)	52 (40–57) (*n* = 13)	0.323
KPS change			0.715
Increase	2 (6.5)	10 (19.6)	
Decrease	2 (6.5)	1 (2.0)	
No change	27 (87.1)	40 (78.4)

**Fig. 1:**
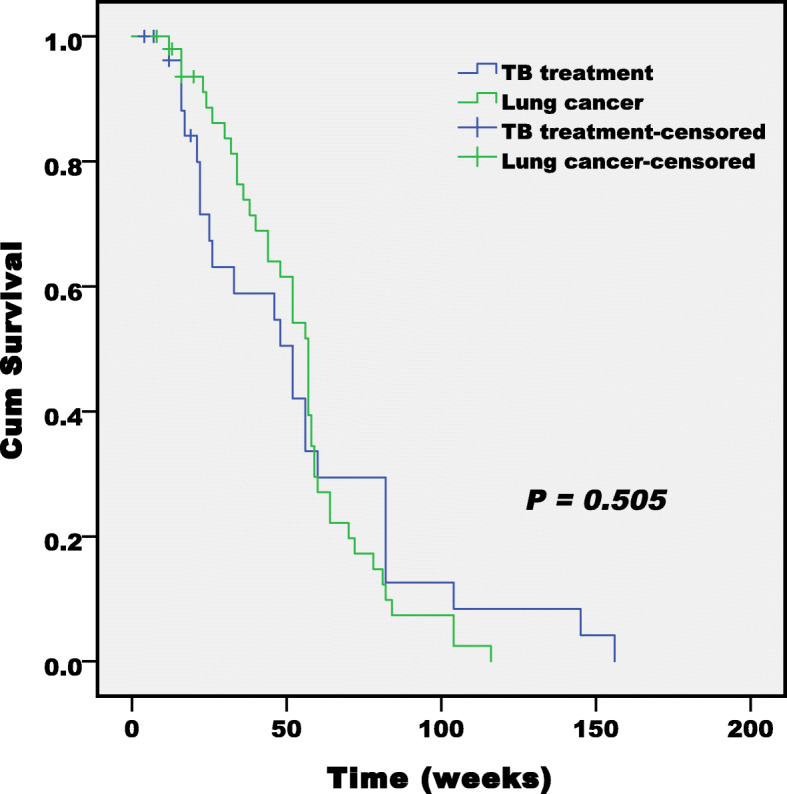
Comparison of Kaplan-Meier curves of survival of two groups

### Clinical course and response to anti-tuberculosis treatments (Table 3)

Active TB was mainly diagnosed by sputum smear (61.3%); the others were diagnosed by sputum culture (38.7%). The majority of active TB patients were new cases (96.8%). 70.9% of patients in the TB treatment group received the anti-tuberculosis treatment at the same time with or after anti-cancer chemotherapy. The 6HRE treatment regimen was the most commonly adopted (74.2%). 6 patients received the 9HR treatment regimen because of liver/renal injury at baseline. During the anti-tuberculosis treatment, treatment interruption occurred in 2 patients, active withdrawal occurred in 4 patients (not due to side effect of treatment), and drug replacement occurred in 3 patients (rifampicin was replaced by rifapentin). Average duration of concurrent chemotherapy was about 3.4 months. The treatment success rate (including cured and treatment completed patients) was 80.7%.

**Table 3:** Comparison of TB characteristics and therapies

variable	TB treatment n (%)
Diagnostic method	
Sputum smear	19 (61.3)
Sputum culture	12 (38.7)
TB patients classification	
New patients	30 (96.8)
Previously treated patients	1 (3.2)
The time of anti-TB treatment	
before anti-cancer chemotherapy	9 (29.0)
at the same time	17 (54.8)
after anti-cancer chemotherapy	5 (16.1)
Anti-TB treatment regimen	
2HRZE/4HR	3 (9.7)
6HRE	23 (74.2)
9HR	5 (16.1)
Duration of concurrent chemotherapy (months)	3.4 ± 1.5
Treatment completion rate	
Completion	25 (80.6)
Delay completion	2 (6.5)
Active withdrawal	4 (12.9)
Treatment outcome	
Cured	18 (58.1)
Treatment completed	7 (22.6)
Treatment failed	2 (6.5)
Died	0 (0)
Lost to follow-up	4 (12.9)

### Side effects of treatments (Table 4)

Side effects of treatments between two groups were not different. The most common adverse effect in TB treatment group was liver injury (61.3%). The most serious side effect in TB treatment patients was leukocyte deficiency (9.7% in Grade 3). Patients receiving granulocyte colony stimulating factor were not different between two groups (5 patients in the TB treatment group and 6 patients in the lung cancer group, *p* = 0.574). No serious adverse events were found relevant to anti-tuberculosis treatments. Rare side effects of anti-tuberculosis treatments, for example peripheral neuropathy, sight damage and skin rash, were not observed in our patients.

**Table 4:** Comparison of side effects of therapies between groups

variable	TB treatment n (%)	Lung cancer n (%)	P value
Leukocyte deficiency			0.887
Nothing	13 (41.9)	26 (51.0)	
Grade 1	7 (22.6)	10 (19.6)	
Grade 2	8 (25.8)	11 (21.6)	
Grade 3	3 (9.7)	4 (7.8)	
Thrombocytopenia			0.508
Nothing	19 (61.3)	37 (72.5)	
Grade 1	6 (19.4)	5 (9.8)	
Grade 2	4 (12.9)	5 (9.8)	
Grade 3	2 (6.5)	2 (3.9)	
Grade 4	0 (0)	2 (3.9)	
Renal toxic effects			0.236
Nothing	15 (48.4)	27 (52.9)	
Grade 1	14 (45.2)	24 (47.1)	
Grade 2	2 (6.5)	0 (0)	
Liver toxicity			0.173
Nothing	12 (38.7)	24 (47.1)	
Grade 1	12 (38.7)	23 (45.1)	
Grade 2	5 (16.1)	4 (7.8)	
Grade 3	2 (6.5)	0 (0)	
Gastrointestinal toxicity			0.067
Nothing	15 (48.4)	32 (62.8)	
Grade 1	9 (29.0)	13 (25.5)	
Grade 2	7 (22.6)	5 (9.8)	
Grade 3	0 (0)	1 (2.0)	
Cardiac toxic effects	6 (19.4)	7 (13.7)	0.499
Neurotoxicity	0 (0)	0 (0)	1

## Discussion

The main finding of the current study is that patients with coexisting lung cancer and active tuberculosis could safely receive both anti-cancer and anti-tuberculosis treatments. This information will help physicians make clinical management decisions for patients with coexisting lung cancer and active tuberculosis.

Lung cancer and tuberculosis are two major public health problems in China. It was demonstrated that there were 28.49 lung cancer-related deaths per 100,000 population in 2014 [1] and 2.6 tuberculosis-related deaths per 100,000 population in 2017 in China [2]. Meanwhile, lung cancer and tuberculosis have a complicated relationship, which means that they are risk factors for each other. As a tuberculosis high-burden country, the incidence of tuberculosis in cancer patients was as high as 12.72% in our past research [16]. In the current study, the features of patients were elderly with advanced NSCLC. In addition, most patients in the TB treatment group were newly diagnosed. Hence, caution should be paid to the risk of tuberculosis in lung cancer patients.

Tuberculosis treatment in cancer patients is still not conclusive, especially for advanced non-small cell lung cancer patients with synchronous anti-tuberculosis and anti-cancer treatments [17]. Kim et al. showed that in cancer patients (lung cancer patients accounted for 8% of subjects), anticancer chemotherapy is not an obstacle to treating tuberculosis [7]. Hirashima et al demonstrated that in patients with metastatic colorectal cancer, both cancer chemotherapy and tuberculosis treatment could be concurrently administered safely and efficiently [8]. The scholar furtherly demonstrated that anti-cancer and anti-tuberculosis treatments can be safely and effectively administered in patients with different types of malignancies (including lung cancer) and active TB [9]. In our study, most patients received the 6HRE treatment regimen (74%), 2 patients received the 2HRZE/4HR treatment regimen and 6 patients received the 9HR treatment regimen because of liver/renal injury at baseline. The TB treatment success rate was as high as 80.7%, just a little lower than that of new and relapse cases in China in 2017 [2]. On the other hand, no serious adverse effects related to anti-tuberculosis treatments were encountered. The most common side effect in TB treatment group was liver injury, most of which were non-serious and recovered by appropriate intervention. The results suggested that anti-tuberculosis treatments would be able to perform in lung cancer patients with active TB.

In our study the sum of rate of TB treatment failure and lost to follow-up reached nearly 20%. The anti-tuberculosis treatment regimen adjustment and withdrawal were probably accounted for the failure (regimen adjustment in 5 patients, treatment interruption in 2 patients, and active withdrawal in 4 patients). The other reason may be because of MRT-TB/RR-TB. Due to limited resources, drug susceptibility testing to TB was performed in 3% of new cases in China in 2013 [18]. MRT-TB/RR-TB was not routinely tested during the study period. Hence, more caution should be paid to the MRT-TB/RR-TB infection in lung cancer patient.

Our study focused on lung cancer patients with active TB, not latent TB. Screening and treatment of latent TB nowadays becomes a new priority action for eliminating TB strategy in high-risk population. The prevalence of latent TB diagnosed by interferon-gamma release assay in China ranged between 13 and 20% in a population-based study [19]. Due to large size of latent TB population, latent TB treatment is not systemically adopted in national guideline, but restricted to patients with diverse immune deficiencies, such as HIV, silicosis, receiving anti-tumour necrosis factor treatment, receiving dialysis, organ or hematologic transplantation [20, 21]. Because of simpler treatment regimen for latent TB compared with active TB, preventive chemotherapy of latent TB may be preferable in lung cancer patients. However, the cost-efficacy of preventive treatment of latent TB in lung cancer patients deserves further research.

As for anti-cancer treatment, no differences were found between two groups, such as treatment regimen, response rate, median survival time, change in KPS and serious side effect. The result implied that anti-tuberculosis treatment did not interfere with anti-cancer treatment in lung cancer patients with active TB. The median survival time in TB treatment patients was 52 weeks, which was shorter than that in Shanghai (16 months in stage III/IV non-small cell lung cancer) [22]. This may be because the difference in economic and medical level between two regions. Therefore, our findings suggest that both anti-cancer and anti-tuberculosis treatments could be safely and effectively administered in advanced lung cancer patients with tuberculosis.

Nowadays cancer immunotherapy, such as immune checkpoint inhibitors (ICBs) targeting cytotoxic T-lymphocyte associated antigen 4, programmed cell death 1 (PD-1), and programmed death ligand 1 (PD-L1), has revolutionized the treatments of a variety of different cancers, including lung cancer. However, TB infection associated with these agents has been increasingly reported [23, 24]. The mechanisms of TB activation after ICBs treatments are not yet defined. Boosting Th1-mediated inflammatory responses with PD-1 blockade, administrating steroids or anti-tumor necrosis factor-alpha agents to overcome immune-related adverse events and lymphopenia are among the potential mechanisms [25, 26]. Hence, more attention should be paid to the TB infection during ICBs treatment. Latent TB screening and chemoprophylaxis before checkpoint therapy may be indicated to prevent active TB.

### Limitations

First, this was a retrospective study with inevitable selection bias. Second, the sample size was a small cohort of patients, limiting the power of the statistical analysis. Third, patients receiving molecular targeted therapy were excluded because there were no cases for analysis. Fourth, due to limited resources, MRT-TB/RR-TB was not tested in our study. Fifth, non-tuberculous mycobacteria could not be completely ruled out from our patients because of the limitations of sputum smear and/or culture. However, the influence is small because anti-Tb treatments failed in only two patients.

## Conclusion

Our results indicate that both anti-cancer and anti-tuberculosis treatments can be safely and effectively administered in lung cancer patients with tuberculosis, and attention should be paid to the risk of tuberculosis in lung cancer patients in a tuberculosis high-burden country.

## Data Availability

The datasets used and/or analyzed during the current study are available from the corresponding author on reasonable request.

## Abbreviations

TB: Tuberculosis
BMI: Body mass index
ESR: Erythrocyte sedimentation rate
ICBs: Immune checkpoint inhibitors
PD-1: Programmed cell death 1
PD-L1: Programmed death ligand 1.

## References

[1] Cao M, Chen W (2019). Epidemiology of lung cancer in China. Thoracic Cancer.

[2] World Health Organization. Global tuberculosis report 2018. Geneva: World Health Organization; 2018. Licence: CC BY-NC-SA 3.0 IGO.

[3] Yu Y-H, Liao C-C, Hsu W-H, Chen H-J, Liao W-C, Muo C-H, Sung F-C, Chen C-Y (2011). Increased lung Cancer risk among patients with pulmonary tuberculosis: a population cohort study. J Thorac Oncol.

[4] Seo GH, Kim MJ, Seo S, Hwang B, Lee E, Yun Y, Choi M, Kim M, Kim JW, Kim ES (2016). Cancer-specific incidence rates of tuberculosis: a 5-year nationwide population-based study in a country with an intermediate tuberculosis burden. Medicine.

[5] Vento S, Lanzafame M (2011). Tuberculosis and cancer: a complex and dangerous liaison. Lancet Oncol.

[6] Simonsen DF, Farkas DK, Horsburgh CR, Thomsen RW, Sørensen HT (2017). Increased risk of active tuberculosis after cancer diagnosis. J Infect.

[7] Kim DK, Lee SW, Yoo C-G, Kim YW, Han SK, Shim Y-S, Yim J-J (2005). Clinical characteristics and treatment responses of tuberculosis in patients with malignancy receiving anticancer chemotherapy. Chest.

[8] Hirashima T, Nagai T, Shigeoka H, Tamura Y, Yoshida H, Kawahara K, Kondoh Y, Sakai K, Hashimoto S, Fujishima M (2014). Comparison of the clinical courses and chemotherapy outcomes in metastatic colorectal cancer patients with and without active Mycobacterium tuberculosis or Mycobacterium kansasiiinfection: a retrospective study. BMC Cancer.

[9] Hirashima T, Tamura Y, Han Y, Hashimoto S, Tanaka A, Shiroyama T, Morishita N, Suzuki H, Okamoto N, Akada S (2018). Efficacy and safety of concurrent anti-Cancer and anti-tuberculosis chemotherapy in Cancer patients with active Mycobacterium tuberculosis: a retrospective study. BMC Cancer.

[10] XY-y CW, Tao L, Hui C (2016). Aanlysis for the global and China TB epidemic situation in 2015. J Tuberc Lung Health.

[11] Department of Disease Control; Department of Medical Administraton (2009). Chinese Center for Disease Control and Prevention: Guidelines for Implementing the National Tuberculosis Control Program in China (2008).

[12] Chinese Society of Clinical Oncology (2018). CSCO Primary Diagnosis and Treatment Guidelines for Lung Cancer.

[13] Eisenhauer EA, Therasse P, Bogaerts J, Schwartz LH, Sargent D, Ford R, Dancey J, Arbuck S, Gwyther S, Mooney M (2009). New response evaluation criteria in solid tumours: revised RECIST guideline (version 1.1). Eur J Cancer.

[14] World Health Organization (2013). A.2.1 Treatment outcomes for TB patients. In: Definitions and reporting framework for tuberculosis.

[15] U.S. Department of Health and Human Services, National Institutes of Health, National Cancer Institute: Common Terminology Criteria for Adverse Events (CTCAE). Version 4.0. Published: May 28, 2009 (v4.03: June 14, 2010).

[16] Chai Mei SQ (2015). Gu kangsheng, : a prevalence study of pulmonary tuberculosis in patients with malignancy in Heifei city. Chin J Dis Control Prev.

[17] Ho JC, Leung CC (2018). Management of co-existent tuberculosis and lung cancer. Lung Cancer.

[18] Islam T, Hiatt T, Hennig C, Nishikiori N (2014). Drug-resistant tuberculosis in the WHO Western Pacific region. Western Pac Surveill Response J.

[19] Gao L, Lu W, Bai L, Wang X, Xu J, Catanzaro A, Cárdenas V, Li X, Yang Y, Du J (2015). Latent tuberculosis infection in rural China: baseline results of a population-based, multicentre, prospective cohort study. Lancet Infect Dis.

[20] World Health Organization. Guidelines on the Management of Latent Tuberculosis Infection. Geneva: © World Health Organization 2015; 2015. 25973515

[21] Cui X, Gao L, Cao B (2020). Management of latent tuberculosis infection in China: exploring solutions suitable for high-burden countries. Int J Infect Dis.

[22] Fan H, Shao Z-Y, Xiao Y-Y, Xie Z-H, Chen W, Xie H, Qin G-Y, Zhao N-Q (2015). Incidence and survival of non-small cell lung cancer in Shanghai: a population-based cohort study. BMJ Open.

[23] Elkington PT, Bateman AC, Thomas GJ, Ottensmeier CH (2018). Implications of tuberculosis reactivation after immune checkpoint inhibition. Am J Respir Crit Care Med.

[24] Barber DL, Sakai S, Kudchadkar RR, Fling SP, Day TA, Vergara JA, Ashkin D, Cheng JH, Lundgren LM, Raabe VN (2019). Tuberculosis following PD-1 blockade for cancer immunotherapy. Sci Transl Med.

[25] Anastasopoulou A, Ziogas DC, Samarkos M, Kirkwood JM, Gogas H (2019). Reactivation of tuberculosis in cancer patients following administration of immune checkpoint inhibitors: current evidence and clinical practice recommendations. J Immunother Cancer.

[26] Reungwetwattana T, Adjei AA (2016). Anti-PD-1 antibody treatment and the development of acute pulmonary tuberculosis. J Thorac Oncol.

